# HFA-PEFF score as a predictor of worsening heart failure after first-time catheter ablation for atrial fibrillation in patients with preclinical heart failure and preserved ejection fraction

**DOI:** 10.3389/fcvm.2025.1704164

**Published:** 2026-01-20

**Authors:** Sho Hirayama, Shinya Fujiki, Ami Maekawa, Soma Sato, Hayao Ikesugi, Kazuyo Tanaka, Yuka Sekiya, Hiroki Tsuchiya, Takayuki Kumaki, Naomasa Suzuki, Ryohei Sakai, Yasuhiro Ikami, Yuki Hasegawa, Sou Otsuki, Hiromi Kayamori, Tsugumi Takayama, Takeshi Kashimura, Takayuki Inomata

**Affiliations:** 1 Department of Cardiovascular and Medicine, Niigata University Graduate School of Medical and Dental Sciences, Niigata, Japan; 2 School of Medicine, Niigata University, Niigata, Japan

**Keywords:** atrial fibrillation, catheter ablation, H_2_FPEF score, HFA-PEFF score, HFpEF

## Abstract

**Background:**

Catheter ablation (CA) is a standard treatment for atrial fibrillation (AF); however, some patients experience worsening heart failure (WHF) afterward. The H_2_FPEF and HFA-PEFF scores are validated tools for HFpEF risk stratification, but their predictive value for WHF after CA in patients with preclinical heart failure (HF) remains unclear.

**Method:**

This retrospective, single-center observational study included 257 AF patients with preserved left ventricular ejection fraction (LVEF) ≥50% and no history or symptoms of HF who underwent first-time CA between February 2017 and September 2022. Patients were classified as high HFpEF score group if they had H_2_FPEF score ≥6 or HFA-PEFF score ≥5. The primary endpoint was WHF: HF hospitalization, initiation of oral diuretics, or intravenous administration of diuretics.

**Results:**

Among 257 patients, 54 (21.01%) were classified as high HFpEF score group. WHF incidence was significantly higher in the high HFpEF score group than in the low HFpEF score group (log-rank *p* < 0.001), while AF recurrence did not differ significantly (log-rank *p* = 0.546). In Firth's penalized logistic regression analysis, high HFA-PEFF score (HR 6.52, 95% CI 1.54–23.21, *p* = 0.014) and AF recurrence (HR 8.18, 95% CI 1.80–77.60, *p* = 0.005) appeared to be potential independent predictors of WHF.

**Conclusions:**

In this exploratory analysis, the HFA-PEFF score potentially represent an independent predictor of WHF after CA in AF patients with preclinical HF and preserved LVEF.

## Introduction

Atrial fibrillation (AF) is the most common arrhythmia, with a growing prevalence worldwide (1). AF is associated with a significant risk of mortality and morbidity, including dementia, stroke, heart failure (HF), and reduced quality of life (2–4). Despite the availability of diverse treatment strategies, effective and individualized management of AF remains a clinical challenge.

Catheter ablation (CA) is widely adopted for rhythm control in patients with AF. While radiofrequency ablation has been the conventional approach, newer techniques such as cryoablation, hot balloon ablation, and pulsed-field ablation are increasingly used in clinical practice (5–7). CA has been routinely performed for approximately three decades, and the number of procedures continues to increase annually (8–10). Importantly, some patients undergoing CA may already be at risk for HF, even if they are in an asymptomatic, preclinical stage. The incidence of newly developed HF after ablation has been reported as 1.1 per 100 patient-year (11). This underscores the need to establish HF risk stratification as part of post-CA management, which has traditionally focused on preventing AF recurrence and the discontinuation of anticoagulant therapy (12–15). Among the various forms of HF, heart failure with preserved ejection fraction (HFpEF) is particularly relevant to risk stratification, as it shares several pathophysiological mechanisms with AF (16, 17). The H_2_FPEF and HFA-PEFF scores are commonly used to diagnose and predict HFpEF (18, 19), yet their utility in predicting HF events after CA remains unclear. In this study, we aimed to evaluate the incidence of WHF events following initial CA for AF and to determine whether HFpEF scoring systems—specifically the H_2_FPEF and HFA-PEFF scores—can aid in post-ablation risk stratification, particularly for identifying patients at risk of *de novo* WHF in the preclinical stage of HF.

## Materials and methods

### Study design and patient population

This single-center, retrospective observational study was conducted at Niigata University Medical and Dental Hospital. We included patients with a left ventricular ejection fraction (LVEF) ≥ 50% who underwent their first-time CA for AF between February 2017 and September 2022. All patients underwent pulmonary vein isolation (PVI) and were followed until November 2024. Eligible patients were considered to be in a preclinical HF, defined by the following criteria: no prior hospitalization for HF, New York Heart Association (NYHA) functional class I, and no use of loop diuretics at the time of admission. The diagnosis of AF was established based on a prior electrocardiogram or 24-hour Holter monitoring. All echocardiographic examinations were performed in the clinical laboratories of our institution within 1 year prior to the CA session. LVEF was assessed using a two-dimensional echocardiographic method. Patients were excluded if they met any of the following criteria: (1) missing key echocardiographic parameters required to calculation of the H_2_FPEF and HFA-PEFF scores—specifically, tricuspid regurgitation pressure gradient, inferior vena cava diameter, or E/e′ ratio, (2) second or subsequent sessions, (3) moderate to severe aortic stenosis, (4) hypertrophic cardiomyopathy, or (5) history of dialysis. Among the excluded patients, only age and sex data were available for comparison, whereas information on AF subtype and heart failure states was not collected. Medical history, comorbidities, medications, and laboratory data were collected from electronic medical records. All procedures were conducted in accordance with the principles of the Declaration of Helsinki and were approved by the institutional ethics committee.

### H_2_FPEF and HFA-PEFF score

The H_2_FPEF score consists of six domains: (1) Heavy (Body mass index >30 kg/m^2^, 2 points), (2) Hypertension (2 or more antihypertensive medicines, 1 point), (3) AF (3 points), (4) Pulmonary Hypertension (Estimated Pulmonary artery systolic pressure >35 mmHg, 1 point), (5) Elder (Age > 60 years, 1 point), and (6) Filling Pressure (E/e′ > 9, 1 point). The total score ranges from 0 to 9, and a score ≥6 is defined as high risk for HFpEF (18). The HFA-PEFF score consists of 3 domains: (1) Functional (septal e′, lateral e′, E/e′, tricuspid regurgitation velocity, or global longitudinal strain), (2) Morphological (left atrial volume index, left ventricular mass index, relative wall thickness, or left ventricular wall thickness), and (3) Biomarker: N-terminal pro-brain natriuretic peptide (NT-proBNP) or brain natriuretic peptide (BNP). The HFA-PEFF score is categorized into major and minor criteria, with each fulfilled major criterion assigned 2 points and each fulfilled minor criterion assigned 1 point. The total score ranges from 0 to 6, with a score of ≥5 indicating a high likelihood of HFpEF (19). These scores were created originally for diagnosis of HFpEF, and it is unknown whether these scores can predict WHF. Both scores have been reported to have similar diagnostic abilities for HFpEF (20, 21); therefore, we combined them and examined the prognostic values for WHF to provide more comprehensive risk assessment. In this study, patients were classified into high HFpEF score group if they had either the H_2_FPEF score ≥6 or the HFA-PEFF score ≥5. The cutoff values for each score were based on the original definitions.

### Procedure details of catheter ablation for atrial fibrillation

PVI or extended PVI was performed in all patients using either cryoballoon, radiofrequency ablation, or hot balloon ablation. Additional procedures—including left atrial posterior wall isolation, superior vena cava isolation, and cavotricuspid isthmus ablation—were performed at the discretion of the operator. Procedure-related complications resulting in delayed hospital discharge occurred in 9 patients. These included one case of access site bleeding, one retroperitoneal hematoma, four cases requiring permanent pacemaker implantation due to sick sinus syndrome, and three cases of cardiac tamponade requiring pericardial drainage. No procedure-related deaths occurred during the study period.

### Study outcome

The primary outcome was WHF including follow events, defined by any of the following events: HF hospitalization, initiation of oral diuretics, or intravenous administration of diuretics (22–24). The oral diuretics include loop and thiazide diuretics. The initiation of thiazide diuretics was defined as WHF only when prescribed as diuretics therapy to relieve congestion and not for antihypertensive treatment. The secondary outcome was the AF recurrence or other supraventricular arrythmias. AF recurrence was defined as any documented episode detected on a 12-lead electrocardiogram, 24-hour Holter monitor, or implantable devices (permanent pacemaker, implantable cardioverter-defibrillator, or cardiac resynchronization therapy devices) after the 3-month blanking period. For 24-hour Holter and device monitoring, AF episodes lasting ≥30 s were considered clinically significant. All events were corrected from the medical records.

### Statistical analysis

Patients were categorized into two groups based on HFpEF risk: high HFpEF score group and low HFpEF score group, using the H_2_FPEF and HFA-PEFF criteria. Continuous variables were presented as mean ± standard deviation (SD) for normally distributed data or number (%). Group comparisons were performed using the Student's *t*-test for normally distributed data or the Mann–Whitney *U* test for non-normally distributed data. There were no missing data in the variables used for the analysis. Time-to-event outcomes including WHF and AF recurrence were assessed using Kaplan–Meier survival analysis, and differences between groups were evaluated with the log-rank test. In univariate regression analysis using Firth's penalized method, the following variables were examined: high H_2_FPEF score, high HFA-PEFF score, AF recurrence, age, male sex, hypertention, diabetes mellitus, dyslipidemia, ischemic heart disease, beta-blockers, angiotensin-converting enzyme inhibitors (ACEi) or angiotensin II receptor blockers (ARBs), angiotensin receptor neprilysin inhibitor (ARNI), MRAs, SGLT2i, hemoglobin (Hb), and creatinine (Cr). The variance inflation factor between the H_2_FPEF and HFA-PEFF scores was 1.1. In multivariate regression analysis using Firth's penalized method, high HFA-PEFF score and AF recurrence were included as independent variables. The sensitivity analysis including age, sex, HFA-PEFF score and AF recurrence were performed by Firth's penalized regression. Receiver operating characteristic (ROC) curve analysis was performed to assess the predictive performance of the H_2_FPEF and HFA-PEFF scores for WHF. Statistical significance was defined as *p* < 0.05. All statistical analyses were performed using JMP version 17 (SAS Institute, Cary, NC, USA) and R software version 4.4.2 (R Foundation, Vienna, Austria).

## Results

### Participants and baseline characteristics

**Figure 1:**
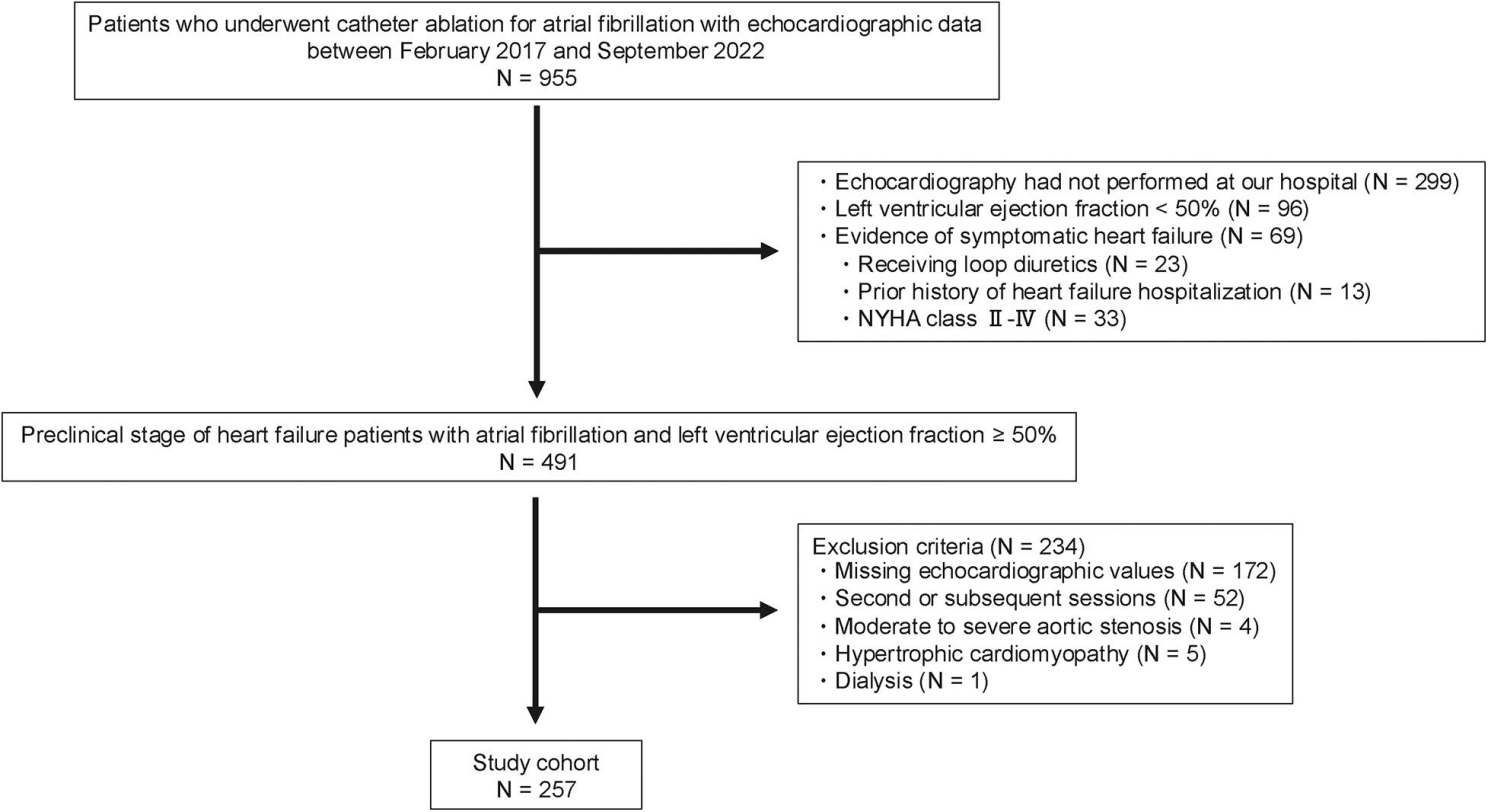
Flowchart of patient inclusion and exclusion in this study. NYHA, New York Heart Association.

**Figure 2:**
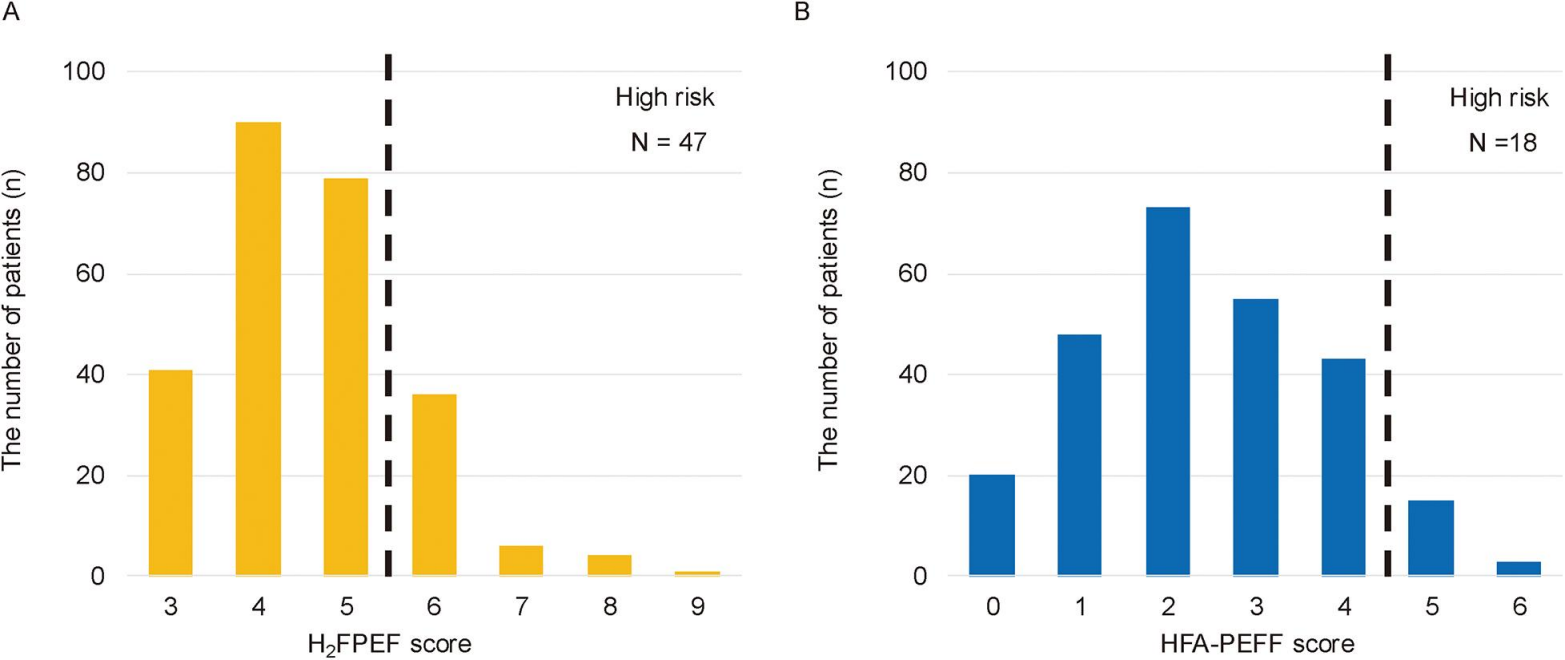
The distribution of patients by H_2_FPEF or HFA-PEFF scores. The 47 patients (18.29%) were classified into the high HFpEF score group based on the H_2_FPEF score ≥6 **(A)**, and the 18 patients (7.00%) based on the HFA-PEFF score ≥5 **(B)** The eleven patients overlapped between the two groups; therefore, a total of the 54 patients were categorized as the high HFpEF score group.

Among the 955 patients who underwent first-time CA for AF between February 2017 and September 2022, 299 were excluded due to echocardiographic examinations conducted at outside facilities. An additional 96 patients were excluded due to a left ventricular ejection fraction (LVEF) < 50%, and 69 patients were excluded because of the evidence of symptomatic HF. Of the remaining 491 patients with preclinical HF, 234 were further excluded due to missing echocardiographic parameters (e.g., tricuspid regurgitation pressure gradient, inferior vena cava diameter, or E/e′ ratio; *n* = 172), second or subsequent CA sessions (*n* = 52), moderate to severe aortic stenosis (*n* = 4), hypertrophic cardiomyopathy (*n* = 5), or dialysis (*n* = 1). Finally, 257 patients were included in the analysis (Figure 1). Of these, 54 (21.01%) were classified into the high HFpEF score group and 203 into the low HFpEF score group based on HFpEF risk scores (Figure 2).

Baseline patient characteristics are summarized in Table 1. The overall mean age was 65 ± 10 years, 69.65% of patients were male, and the mean LVEF was 65.29 ± 6.06%. Compared with the low HFpEF score group, the high HFpEF score group was older (71 vs. 64 years, *p* < 0.001) and had a lower proportion of males (48.15% vs. 75.37%, *p* < 0.001). In addition, the high HFpEF score group had lower Hb levels (13.64 ± 1.43 vs. 14.30 ± 1.40 g/dL, *p* = 0.003), higher BNP levels (111.26 ± 93.61 vs. 62.30 ± 61.10 pg/mL, *p* < 0.001), and higher usage rates of beta-blockers (66.77% vs. 49.75%, *p* = 0.025), ACEi or ARBs (59.26% vs. 28.08%, *p* < 0.001). There were no significant differences between the groups in LVEF (*p* = 0.258) or AF pattern (paroxysmal vs. persistent, *p* = 0.549). There were no significant differences between the included and excluded patients (65 ± 10 vs. 64 ± 11 years, *p* = 0.095), or sex (male: 69.65% vs. 71.38%, *p* = 0.650).

**Table 1:** Patients characteristics.

Baseline characteristics	Total *N* = 257	Low HFpEF score group *N* = 203	High HFpEF score group *N* = 54	*p* value
Age, years	65 ± 10	64 ± 11	71 ± 6	<0.001
Male, *n* (%)	179 (69.65)	153 (75.37)	26 (48.15)	<0.001
Height, cm	165.78 ± 9.39	167.11 ± 8.91	160.79 ± 9.54	<0.001
Weight, kg	64.95 ± 11.99	65.33 ± 11.45	63.53 ± 13.86	0.327
BMI, kg/cm^2^	23.53 ± 3.42	23.27 ± 2.94	24.52 ± 4.72	0.090
Medical history				
Persistent atrial fibrillation, *n* (%)	91 (35.41)	70 (34.48)	21 (38.89)	0.549
Hypertension, *n* (%)	131 (50.97)	89 (43.84)	42 (77.78)	<0.001
Diabetes mellitus, *n* (%)	32 (12.45)	22 (10.84)	10 (18.52)	0.145
Dyslipidemia, *n* (%)	71 (27.63)	53 (26.11)	18 (33.33)	0.298
Ischemia heart disease, *n* (%)	14 (5.45)	9 (4.43)	5 (9.26)	0.192
Clinical score				
CHADS_2_ score	1.21 ± 1.10	1.12 ± 1.14	1.52 ± 0.84	0.001
CHA_2_DS_2_VAsc score	2.09 ± 1.48	1.89 ± 1.46	2.85 ± 1.32	<0.001
HAS-BLED score	0.97 ± 0.89	0.91 ± 0.92	1.20 ± 0.74	0.003
H_2_FPEF score	4.58 ± 1.12	4.16 ± 0.73	6.17 ± 0.86	<0.001
HFA-PEFF score	2.3 ± 1.38	2.06 ± 1.19	3.80 ± 1.22	<0.001
Laboratory date				
Hb, g/dL	14.16 ± 1.43	14.30 ± 1.40	13.64 ± 1.43	0.003
Cr, mg/dL	0.84 ± 0.18	0.84 ± 0.17	0.84 ± 0.21	0.790
BNP, pg/mL	72.59 ± 71.76	62.30 ± 61.10	111.26 ± 93.61	<0.001
Echocardiography				
Heart rate, bpm	72.22 ± 17.83	72.77 ± 18.29	70.17 ± 15.99	0.424
LVDd, mm	46.72 ± 4.39	46.82 ± 4.38	46.35 ± 4.47	0.489
LVDs, mm	29.96 ± 3.85	30.10 ± 3.85	29.46 ± 3.82	0.243
LVEF, %	65.29 ± 6.06	65.06 ± 6.19	66.14 ± 5.53	0.258
IVST, mm	9.38 ± 1.31	9.31 ± 1.29	9.64 ± 1.36	0.099
PWT, mm	9.07 ± 1.08	8.99 ± 1.07	9.36 ± 1.09	0.025
septal e′, m/s	8.45 ± 2.21	8.78 ± 2.34	7.31 ± 1.11	0.063
E/E′	8.09 ± 2.39	7.33 ± 1.72	10.74 ± 2.59	<0.001
LAVI, mL/m^2^	41.50 ± 15.51	39.73 ± 14.58	48.09 ± 17.20	<0.001
TRV, m/s	1.85 ± 0.83	1.74 ± 0.90	2.22 ± 0.25	0.103
Drug				
Beta-blockers, *n* (%)	137 (53.31)	101 (49.75)	36 (66.67)	0.025
ACE inhibitors or ARBs, *n* (%)	89 (34.63)	57 (28.08)	32 (59.26)	<0.001
ARNI, *n* (%)	1 (0.39)	1 (0.49)	0 (0.00)	0.492
MRAs, *n* (%)	6 (2.34)	3 (1.48)	3 (5.56)	0.112
SGLT2 inhibitors, *n* (%)	4 (1.56)	3 (1.48)	1 (1.85)	0.847

ACEi, angiotensin converting enzyme inhibitors; ARBs, angiotensin Ⅱ receptor blockers; ARNI, angiotensin receptor neprilysin inhibitor; BMI, body mass index; BNP, brain natriuretic peptide; Cr, creatinine; Hb, hemoglobin; HFpEF, heart failure with preserved ejection fraction; IVST, intraventricular septum thickness; LAVI, left atrial volume index; LVDd, left ventricular end-diastolic diameter; LVDs, left ventricular end-systolic diameter; LVEF, left ventricular ejection fraction; MRAs, mineralocorticoid receptor antagonists; PWT, posterior wall thickness; SGLT2i, sodium-glucose cotransporter 2 inhibitors; TRV, tricuspid regurgitant velocity. Data are represented as mean ± standard deviation or number (%). *P* values were compared with low HFpEF score and high HFpEF score groups.

### Incidence of worsening heart failure: primary endpoint analysis

**Figure 3:**
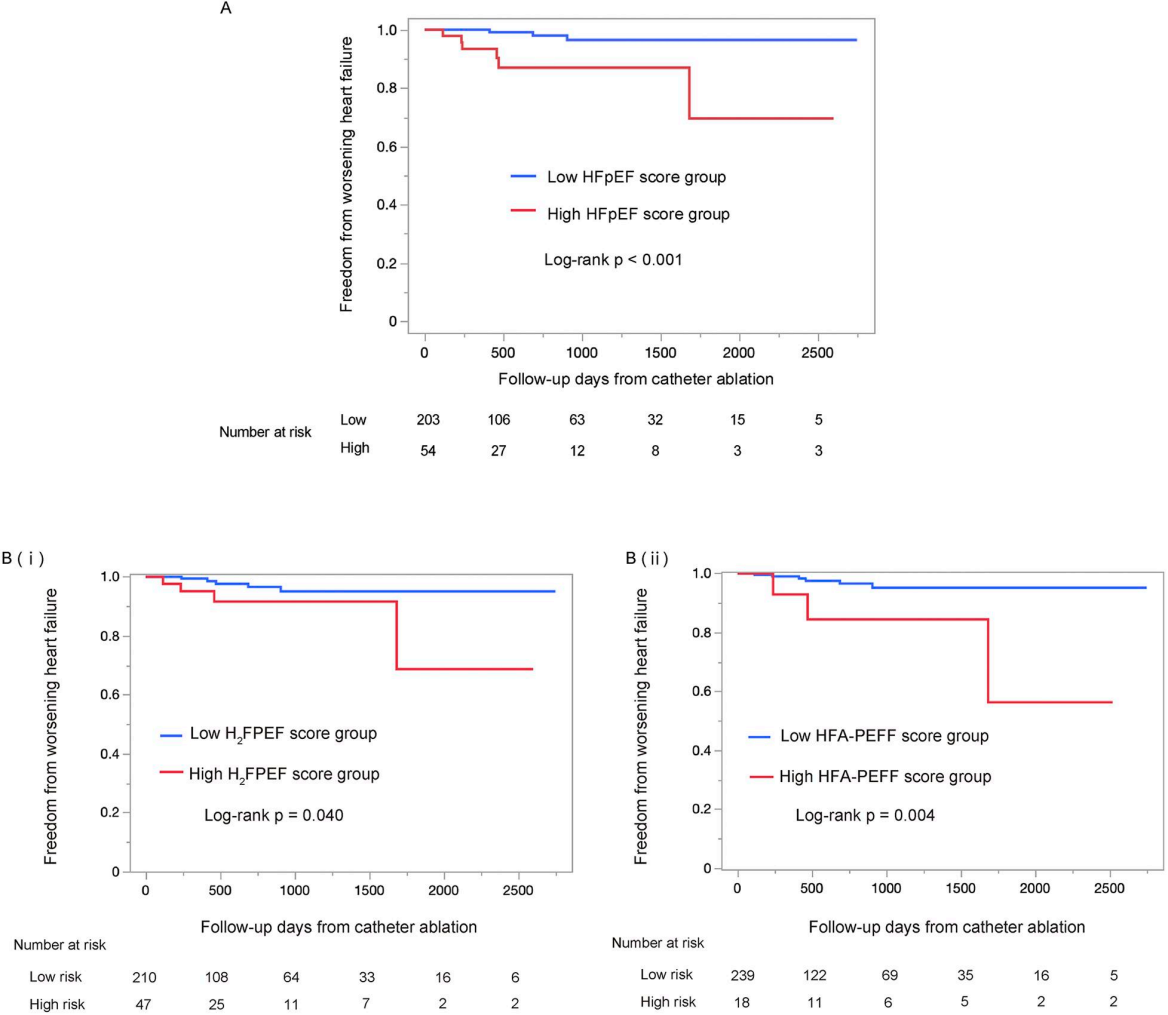
Kaplan–Meier survival analysis of the worsening heart failure between the low HFpEF score and the high HFpEF score groups, using the log-rank test. The high HFpEF score group was defined as patients who had either the H_2_FPEF score ≥6 or the HFA-PEFF score ≥5 **(A)**. The analysis using each score individually are shown in **(B)** (i) and (ii), respectively. The high HFpEF score group was defined as H_2_FPEF score ≥6 (i) or HFA-PEFF score ≥5 (ii). HFpEF, heart failure with preserved ejection fraction.

The median follow-up period was 557 days (interquartile range: 275–1,264 days), during which 9 WHF events occurred. WHF events were significantly more frequent in the high HFpEF score group than in the low HFpEF score group (log-rank *p* < 0.001; Figure 3A). This trend remained consistent when patients were stratified by either the H_2_FPEF score or the HFA-PEFF score individually [log-rank *p* = 0.040; Figure 3B(i), log-rank *p* = 0.004; Figure 3B(ii)]. In the low HFpEF score group, there were two initiations of oral diuretics, and one HF hospitalization. In contrast, in the high HFpEF score group, there were four initiations of oral diuretics, one intravenous admission of diuretics, and one HF hospitalization. No heart failure related deaths occurred during the follow-up period in either group. Detailed information on WHF events is provided in Supplementary Tables S1, S2.

### Atrial fibrillation recurrence rates: secondary endpoint analysis

**Figure 4:**
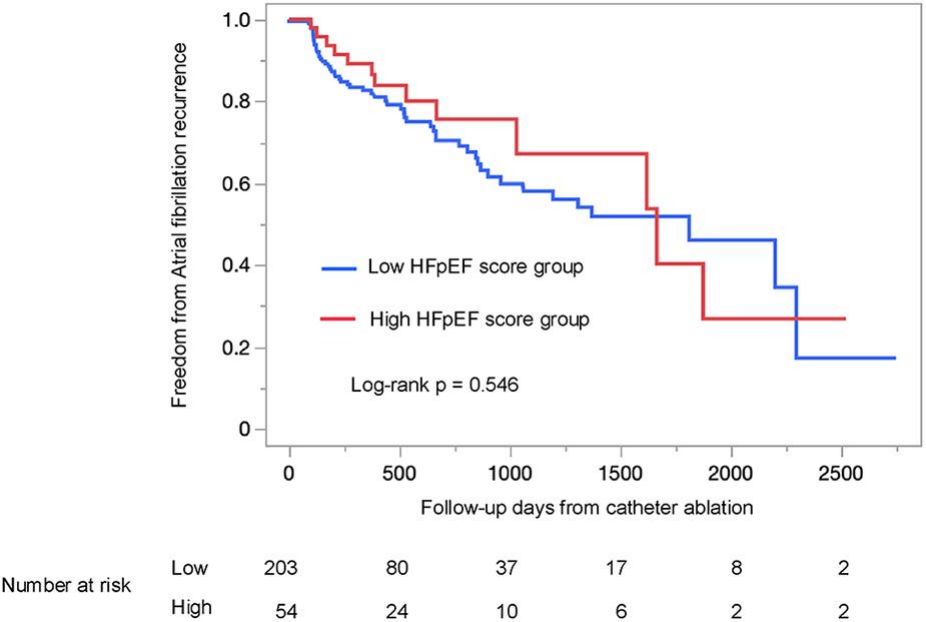
Kaplan–Meier survival analysis of atrial fibrillation recurrence between the low HFpEF score and the high HFpEF score groups, using the log-rank test. The high HFpEF score group was defined as patients who had either the H_2_FPEF score ≥6 or the HFA-PEFF score ≥5. HFpEF, heart failure with preserved ejection fraction.

The AF recurrence rate did not significantly differ between the two groups (log-rank *p* = 0.546; Figure 4). This trend remained consistent when patients were stratified by either the H_2_FPEF score or the HFA-PEFF score individually (log-rank *p* = 0.597; Supplementary Figure S1A, log-rank *p* = 0.639; Supplementary Figure S1B).

### Risk factors for worsening heart failure

In univariable regression analysis using Firth's penalized method, both the high HFA-PEFF score and AF recurrence were significantly associated with WHF (Table 2). In multivariate analysis using Firth's penalized method, the high HFA-PEFF score and AF recurrence remained to be potential independent predictors of WHF (HR 6.52, 95% CI 1.54–23.21, *p* = 0.014; HR 8.18, 95% CI 1.80–77.60, *p* = 0.005, respectively). In addition, the sensitivity analysis using Firth's penalized regression was performed including age, sex, high HFA-PEFF score, and AF recurrence. The High HFA-PEFF score and AF recurrence remained significantly associated with WHF (HR 7.14, 95% CI 1.50–32.06, *p* = 0.015; HR 7.79, 95% CI 1.71–74.06, *p* = 0.006, respectively), while age and sex were not significant (Supplementary Table S3).

**Table 2:** Firth's penalized regression analysis for worsening heart failure after first-time catheter ablation for atrial fibrillation.

Covariates	Univariate			Multivariate		
	HR	95% CI	*p* value	HR	95% CI	*p* value
High H_2_FPEF score	3.71	(0.99–13.14)	0.051			
High HFA-PEFF score	6.66	(1.57–23.67)	0.013	6.52	(1.54–23.21)	0.014
AF recurrence	8.29	(1.82–78.68)	0.005	8.18	(1.80–77.60)	0.005
Age	1.00	(0.94–1.07)	0.985			
Male	0.63	(0.18–2.65)	0.476			
Hypertension	1.15	(0.30–5.47)	0.827			
Diabetes mellitus	1.58	(0.17–7.08)	0.627			
Dyslipidemia	2.10	(0.56–7.41)	0.254			
Ischemic heart disease	1.11	(0.01–8.87)	0.946			
Beta-blockers	0.31	(0.06–1.16)	0.084			
ACE inhibitors or ARBs	2.07	(0.59–7.74)	0.252			
ARNI	12.66	(0.10–107.93)	0.208			
MRAs	6.00	(0.63–27.49)	0.102			
SGLT2 inhibitors	5.92	(0.05–50.30)	0.337			
Hb	1.22	(0.76–1.98)	0.414			
Cr	0.27	(0.01–5.11)	0.472			

ACEi, angiotensin converting enzyme inhibitors; AF, atrial fibrillation; ARBs, angiotensinⅡreceptor blockers; ARNI, angiotensin receptor neprilysin inhibitor; CI, confidence interval; Cr, creatinine; Hb, hemoglobin; HFpEF, heart failure with preserved ejection fraction; HR, hazard ratio; MRAs, mineralocorticoid receptor antagonists; SGLT2i, sodium-glucose cotransporter 2 inhibitors.

### Receiver operating characteristics analysis for predicting worsening heart failure of the H_2_FPEF and HFA-PEFF scores

**Figure 5:**
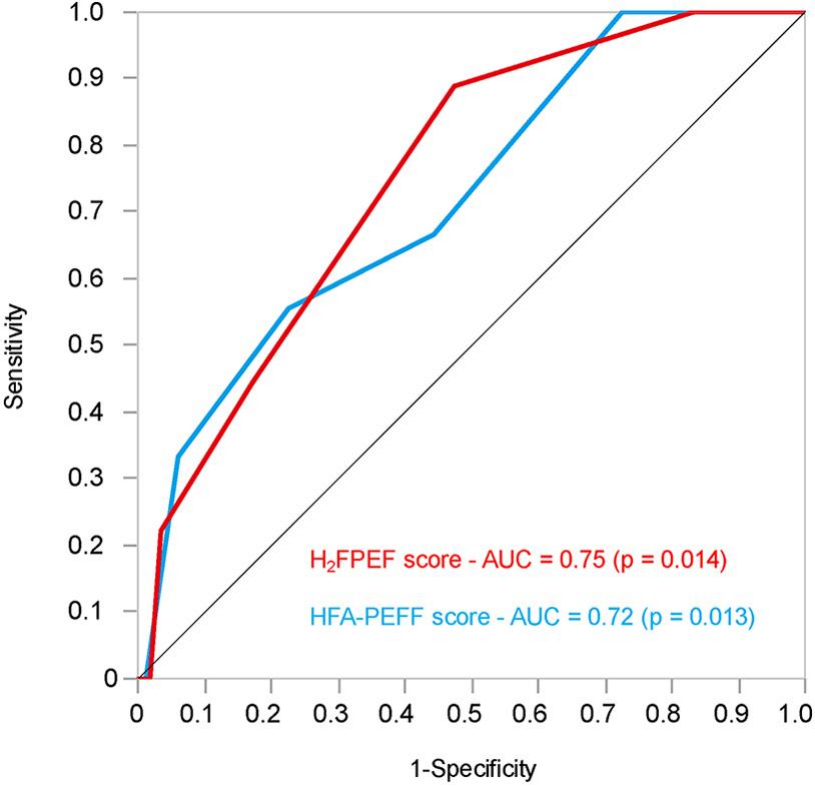
The receiver operating characteristics analysis of the H_2_FPEF and HFA-PEFF scores to predict the worsening heart failure after first-time catheter ablation for atrial fibrillation in patients with preserved ejection fraction. AUC, area under the curve.

To evaluate the predictive performance of the H_2_FPEF and HFA-PEFF scores for WHF after CA for AF, receiver operating characteristic (ROC) analysis was performed (Figure 5). The area under the curve (AUC) values for the H_2_FPEF and HFA-PEFF scores were 0.75 (*p* = 0.014) and 0.72 (*p* = 0.013), respectively. An H_2_FPEF score ≥6 yielded a sensitivity of 44.44% and specificity of 82.66%, while an HFA-PEFF score ≥5 yielded a sensitivity of 33.33% and specificity of 94.00%. The optimal cutoff values for predicting WHF after first-time CA for AF were 5 for the H_2_FPEF score (sensitivity 88.89%, specificity 52.42%) and 4 for HFA-PEFF score (sensitivity 55.66%, specificity 77.42%).

## Discussion

This study revealed the following five findings: (1) Among asymptomatic patients with preclinical HF and preserved LVEF who underwent CA for AF, approximately 20% were classified as high risk for HFpEF according to either of the two risk scores systems; (2) Patients in the high HFpEF score group had a significantly higher incidence of WHF than those in the low score group; (3) There was no significant difference in AF recurrence between the two groups; (4) A high HFA-PEFF score and AF recurrence were identified as independent risk factors of WHF in multivariate analysis, and (5) The most appropriate cut-off values for predicting WHF after first-time CA for AF were 5 for the H_2_FPEF score and 4 for the HFA-PEFF score in this study. These results may suggest that evaluating HFpEF risk in patients with preclinical HF undergoing first-time CA for AF is important, regardless of procedural success. Moreover, when using these scores for risk stratification, alternative cutoff values may be more appropriate for predicting WHF in this population.

### Non-negligible incidence of worsening heart failure events

In this study, nearly 20% of preclinical HF patients with preserved LVEF who underwent CA for AF were classified as high risk for HFpEF, and WHF events occurred in 9 out of 257 patients (3.50%) during the follow-up period. The study population corresponds to stage A or B HF, and previous reports indicate that 1%–6% of such patients progress to stage C or D within 4 years (25). However, limited evidence exists regarding HF progression in patients with stage A or B who have preserved LVEF and coexisting AF. Our finding—that a substantial proportion of asymptomatic AF patients were stratified as high risk, and that a non-negligible number subsequently developed WHF—suggests underlying vulnerability in this population. AF contributes to HF development and progression via mechanisms such as impaired atrial contractility and structural remodeling, including left atrial fibrosis and dilation (16). Conversely, HF can promote AF through elevated sympathetic activity, increased left ventricular filling pressure (26), atrial remodeling and dysfunction (27). These bidirectional interactions suggest a mutually reinforcing pathophysiological relationship between AF and HF. In addition, the coexistence of AF and HFpEF is associated with poor prognosis, including higher mortality and increased risk of WHF (28). Thus, in pre-clinical HF patients with AF, it is important to evaluate atrial and diastolic function (29). In our study, high HFpEF score group experienced a higher incidence of WHF after first-time CA for AF. These findings suggest that some patients with AF-related HF may already have a subclinical but advanced HF state prior to CA—one that CA alone may not fully address. Several previous studies have validated the prognostic value of HFpEF risk scores for HF (30, 31); however, few studies have examined the prognostic values of HFpEF risk scores for *de novo* HF in preclinical HF patients with AF. This study indicated that pre-CA risk stratification using these scores could be useful tools to guide post-CA management for WHF.

### Implications for long-term management of heart failure after catheter ablation

Long-term management after successful CA for AF has become an increasingly important clinical issue. In particular, the prevention of AF recurrence and decisions regarding the continuation of anticoagulation therapy remain topics of ongoing debate. Reported recurrence rates range from 20% to 40% (32), indicating the importance of post CA follow-up. Pre-procedural risk stratification and post-procedural strategies—including corticosteroids, ACEi, and SGLT2i—have been explored to mitigate recurrence (12–14). Although current guidelines do not provide specific recommendations for recurrence prevention, close monitoring is especially important in patients identified as high risk. Established risk factors for AF recurrence include age, left atrial size, duration of AF, and a history of CA (33, 34). However, in this study, HFpEF scores did not predict AF recurrence, suggesting their limited role in recurrence risk assessment.

In this study, we focused on the incidence of WHF after CA. CA for patients with AF and concomitant HF has been generally associated with improved exercise tolerance, increased LVEF, and reduced HF hospitalizations, regardless of baseline LVEF (35–37). These findings suggest that recurrent AF may contribute to the development or WHF. Indeed, in our multivariate analysis, AF recurrence was identified as an independent risk factor for WHF. Careful monitoring is therefore warranted in patients who experience AF recurrence after CA, regardless of their HFpEF risk status. Conversely, in this study, patients classified as high risk by HFpEF scoring systems experienced a higher incidence of WHF events after CA. This finding indicates that WHF can develop independently of AF recurrence, highlighting the need to establish predictive strategies that are distinct from recurrence-based assessments. In addition, only the HFA-PEFF score was identified as an independent predictor of WHF, whereas H_2_FPEF score was not. Although exploratory, the HFA-PEFF score may have better prognostic ability for predicting WHF. This may be because the H_2_FPEF score mainly consists of general risk factors such as body mass index and age, whereas the HFA-PEFF score includes more HF-specific parameters, including echocardiographic indices. Finally, we used the H_2_FPEF and HFA-PEFF scores to stratify HF risk and categorized patients according to previously reported thresholds. However, these thresholds were originally developed for the diagnosis of HFpEF, not for predicting future HF events (18, 19). Receiver operating characteristic (ROC) analysis identified optimal cut-off values of 5 points for the H_2_FPEF score and 4 points for the HFA-PEFF score. These results may suggest that revised thresholds may be more appropriate for predicting WHF after CA; however, the estimates of the AUC and the optimal cut-off values are likely to be unstable because of the small number of WHF events. Therefore, the validation in a larger population is required in future studies.

### Limitation

There are some limitations in this study. First, initiation of thiazide diuretics was included as part of the definition of WHF, although this is not commonly used for this purpose in recent clinical studies. Second, symptomatic patients may not have been completely excluded from the study cohort. Patients with HF who were not taking loop diuretics may have been included. Third, this is a single-center, retrospective observational study with a relatively small cohort size and a limited number of events. The study results, especially those from the Cox regression analysis, may be unstable because age and sex, which were significantly different at the baseline, were not included in the model. Although the results were similar in the sensitivity analysis, the statistical model may have been overfitted and the study results require cautious interpretation. Fourth, the ROC analysis requires cautious interpretation because of the small number of WHF events. Finally, the study data were retrospectively extracted from medical records, which may limit the generalizability of the findings.

## Conclusion

In this exploratory analysis, the HFA-PEFF score potentially represent an independent predictor of WHF after first-time CA for AF in patients with preclinical HF and preserved LVEF (≥50%). This score may be useful for WHF management after CA for AF; however, further validation in a larger population is required before these findings can be applied clinically.

## Data Availability

The datasets presented in this article are not readily available because the deidentified participant data will not be shared. Requests to access the datasets should be directed to Shinya Fujiki, sfujiki@med.niigata-u.ac.jp.
