# Supplementary material for: HFA-PEFF score as a predictor of worsening heart failure after first-time catheter ablation for atrial fibrillation in patients with preclinical heart failure and preserved ejection fraction
Source: Front Cardiovasc Med. 2026 Jan 20;12:1704164. doi: 10.3389/fcvm.2025.1704164 (PMC12864457; doi:10.3389/fcvm.2025.1704164)
Supplement: Supplementary file 1 [file Datasheet1.pdf]

Supplemental Appendix

Supplemental Table 1 Events of worsening heart failure

	Low HFpEF score group	High HFpEF score group
Initiation of oral diuretics	2	4
Intravenous admission of diuretics	0	1
Heart failure hospitalization	1	1
Heart failure related deaths	0	0

The high HFpEF score group was defined as patients who had either the H_2FPEF score ≥ 6 or the HFA-PEFF score ≥ 5. Initiation of oral diuretics was defined as the new prescription of either loop or thiazide diuretics. HFpEF, heart failure with preserved ejection fraction.

Supplemental Table 2   Details of worsening heart failure events

Patients	Age	Sex	Type of event	Days after CA	AF recurrence status	Treatment	HFpEF score group
1	59	M	Oral diuretics	689	N	Medication	L
2	67	M	Oral diuretics	414	PAF	Re-ablation	L
3	67	F	HF Hospitalization	907	PeAF	Re-ablation	L
4	59	F	Oral diuretics	240	N	Medication	H
5	60	M	HF Hospitalization	235	N	Medication	H
6	73	M	Oral diuretics	472	PAF	Re-ablation	H
7	74	F	Oral diuretics	116	N	Medication	H
8	71	F	IV diuretics	1685	PeAF	Re-ablation	H
9	67	M	Oral diuretics	460	N	Medication	H

The high HFpEF score group was defined as patients who had either the H_2FPEF score ≥ 6 or the HFA-PEFF score ≥ 5. Initiation of oral diuretics was defined as the new prescription of either loop or thiazide diuretics. AF, atrial fibrillation; CA, catheter ablation; F, female; H, high HFpEF score group; HF, heart failure; HFpEF, heart failure with preserved ejection fraction; IV, intravenous; L, low HFpEF score group; M, male; N, no recurrence; PAF, paroxysmal atrial fibrillation; PeAF, persistent atrial fibrillation

Supplemental Table 3 Sensitivity analysis adjusted for age and male using penalized Cox regression

	HR	95% CI	p value
High HFA-PEFF score	7.14	(1.50-32.06)	0.015
AF recurrence	7.79	(1.71-74.06)	0.006
Age	0.98	(0.91-1.06)	0.546
Male	0.82	(0.23-3.16)	0.764

AF, atrial fibrillation; CI, confidence interval; HR, heart rate

Supplemental Figure 1

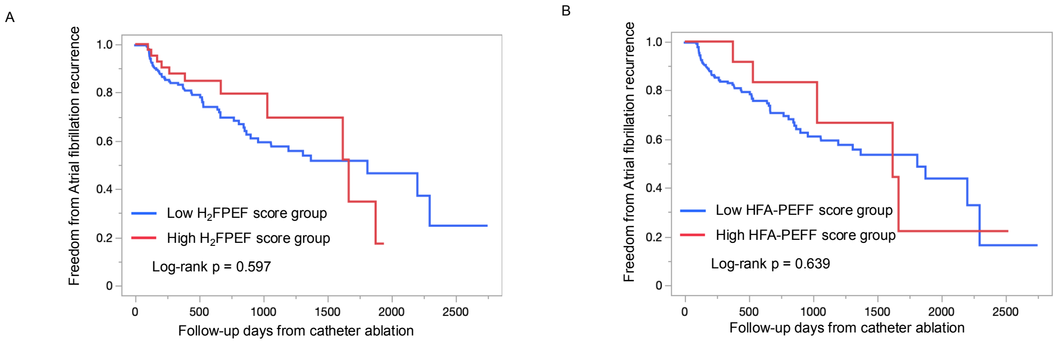

A — Number at risk

Low	210	83	38	18	9	3
High	47	21	9	5		

B — Number at risk

Low	239	92	41	19	8	2
High	18	12	6	4	2	2

Supplemental Figure 1: Kaplan–Meier survival analysis of atrial fibrillation recurrence between the low HFpEF score and the high HFpEF score groups, using the log-rank test, based on the H_2FPEF score (A) and HFA-PEFF score (B). The high HFpEF score group was defined as patients who met the H_2FPEF score ≥ 6 (A) and the HFA-PEFF score ≥ 5 (B). HFpEF, heart failure with preserved ejection fraction.
